# Effect of potential risk factors on renal functions in simultaneous bilateral total knee arthroplasty

**DOI:** 10.3389/fsurg.2024.1405487

**Published:** 2024-08-29

**Authors:** Bedrettin Akar, Fatih Ugur, Mucahid Osman Yucel, Ferhan Aytug

**Affiliations:** ^1^ Deparmant of Orthopedics and Traumatology, Sakarya Yenikent State Hospital, Sakarya, Türkiye; ^2^ Deparmant of Orthopedics and Traumatology, Kastamonu University Faculty of Medicine, Kastamonu, Türkiye; ^3^ Deparmant of Orthopedics and Traumatology, Duzce University Faculty of Medicine, Sakarya, Türkiye

**Keywords:** total knee arthroplasty, glomerular filtration rate, acute kidney injury, postoperative complication, osteoarthritis, knee

## Abstract

**Objective:**

This retrospective study investigated the risk factors leading to a decrease in Renal glomerular filtration rate (eGFR) and the development of acute kidney injury (AKI) during the early postoperative period in patients undergoing simultaneous bilateral total knee arthroplasty (SBTKA).

**Methods:**

SBTKA was performed on 862 patients between 2014 and 2021 in a single center, by a single surgeon. The risk factors affecting the development of AKI were analyzed by monitoring the changes in pre-and postoperative serum creatinine (Scr) levels and eGFR values. RIFLE criteria were used to evaluate the kidney functions of the patients, who were followed up for an average of 6 months.

**Results:**

While there was no decrease in eGFR or AKI in 818 patients postoperatively, eGFR decreased and AKI of different stages developed in 44 patients, according to the RIFLE criteria. Of the 44 patients with AKI, 31 had Risk, 9 had Injury, 3 had Failure, and one had Loss of kidney function. Two patients with American Society of Anaesthesiologists (ASA) class IV died due to deepening of postoperative renal dysfunction.

**Conclusion:**

We found that the direct risk factors in SBTKA in terms of eGFR decrease and AKI development include long operation time, increased need for blood transfusion, and diabetic nephropathy, while increased body mass index (BMI) is an indirect risk factor. When planning for a SBTKA, we presume that a thorough analysis of these factors will decrease AKI risk.

## Introduction

Osteoarthritisis one of the most common pathologies of advanced ages and causes arthrosis in the knees (1–3). Degeneration of the knees causes a decrease in the quality of life of patients due to pain and limitation of movement (3–6). Sixty percent of the patients have bilateral symptoms when presenting to the surgeon, and surgery is usually indicated in both knees (7–9). Options for patients scheduled for bilateral operation include either simultaneous bilateral replacement in a single session, performance of the arthroplasty on the first and second knees 1 week apart in the same hospitalization period or 3 or 6 months apart. Bilateral operation in a single anesthesia session has advantages, such as the ease of rehabilitation, short hospital stays, and cost reduction. Patients who do not want to be hospitalized for the first time and, above all, do not want to experience the risks of surgical pain and complications after the operation for the second time, can prefer SBTKA with the advice of their orthopedist.

SBTKA is a long operation that can adversely affect the patient's hemodynamics (10–13). AKI, a feared complication of this elective surgery, increases mortality and morbidity, prolongs the hospital stay, and may result in invasive needs, such as dialysis (14–17). AKI risk factors in SBTKA surgery include long operative time, increased patient age, preoperative low eGFR, high ASA scores, increased body mass index (BMI), increased need for blood transfusion, comorbidities, anesthesia type, ACE inhibitor use, and male gender (18, 19) (Table 1).

**Table 1:** ASA classification.

ASA class	Definition
I	A normally healthy patient
II	A patient with mild systemic disease
III	A patient with systemic disease which is not incapacitating
IV	A patient with an incapacitating systemic disease that is constant threat to life
V	A moribund patient who is not expected to survive for 24 h with or without operation

ASA, American Society of Anesthesiologists.

## Materials and methods

Between 2014 and 2021, SBTKA was performed on 862 patients with a mean age of 72 (51–92) years in a single center, by a single surgeon. Ethics committee approval was obtained from Sakarya University Faculty of Medicine on 22.03.2021 with document number E.20135-177. Patients who underwent staged BTKA, those with bilateral unicondylar knee prostheses, rheumatoid arthritis, and ankylosing spondylitis, morbidly obese patients, and patients receiving oncological treatment were excluded from the study. The reference range of eGFR in adults is 90–122 ml/min, and it is evaluated together with pre- and postoperative serum creatinine (Scr) levels (18–21). For the assessment of AKI, the Scr value measured within one month preoperatively was considered baseline, and the highest postoperative Scr was considered a sign of maximum AKI. In the power analysis performed with the G*power 3.1 program, the effect size for the presence of AKI in the study and control groups was 0.28 (**Staggered Rather Than Staged or Simultaneous Surgical Strategy May Reduce the Risk of Acute Kidney Injury in Patients Undergoing Bilateral TKA**) (alpha error = 0.05). In the sample size analysis performed with a power value of 0.80, the total number of samples required was 84 (42 patients for each group). Subsequently, randomly selected 58 patients among 818 patients who did not develop AKI were included in the statistical analysis. The two groups were compared in terms of age, eGFR, operation time, obesity, ASA score, and comorbidities. The patients underwent primary arthroplasty under a tourniquet with epidural anesthesia, first on the right and then on the left. The surgical technique was as follows: Appropriate femoral and tibial osteotomies were performed by tilting the patella laterally with an anterior long incision. Cemented knee prostheses of different brands (Wright, Orthopedia, Consensus) that protect the posterior cruciate ligament were used with patellar components in all patients. In both techniques, bleeding was controlled after the tourniquet was opened and the layers were closed anatomically with adrain. The patients were administered prophylactic antibiotics (3 × 1 grams of cephazolin) and anticoagulants (1 × 0.8 ml enoxaparin sodium) one day before the operation, along with antithrombotic socks. The patients were monitored for urine output with a urinary catheter. RIFLE criteria were used to evaluate the kidney functions of the patients followed up for an average of 6 months (Table 2).

**Table 2:** RIFLE criteria.

Class	Glomerular filtration rate	Urine output criteria
Risk-**R**	Serum creatinine × 1.5	< 0.5 ml/kg/h × 6 h
Injury-**I**	Serum creatinine × 2	< 0.5 ml/kg/h × 12 h
Failure-**F**	Serum creatinine × 3, or serum creatinine ≥ 4 mg/dl with an acute rise > 0.5 mg/dl	< 0.3 ml/kg/h × 24 h, or anuria × 12 h
Loss-**L**	Persistent acute renal failure = complete loss of kidney function > 4 weeks	
End-stage kidney disease-**E**	End-stage kidney disease > 3 months	

### Statistical evaluation

Statistical calculations were performed with (Number Cruncher Statistical System) 2007 Statistical Software (Utah, USA) package program for Windows.

Besides descriptive statistical methods (mean, standard deviation), the distribution of the variables was examined with the Shapiro–Wilk test of normality. The variables indicate a normal distribution unpaired *t* test was used in the comparison of groups, the paired *t*-test was used for the pre-and postoperative comparisons, and the Chi-square test was used for the comparison of the qualitative data. Multivariet Logistic Regression analysis was performed to separate the factors affecting the development of AKI. Receiver operating characteristics (ROC) analysis was used to calculate the area under the curve (AUC) for Operation Time (min) and Blood Transfusion (unit) and to find the best Operation Time (min) and Blood Transfusion (unit) cut-off values for identifying the progression to AKI groups. To calculate the sensitivity, specificity, PPV, NPV and LR(+) for the Operation Time (min) and Blood Transfusion (unit) measurements at varying cut-off values, a conventional receiver operating characteristic curve was generated and the area under the curve (AUC) was calculated to Operation Time (min) and Blood Transfusion (unit). The results were evaluated at a significance level of *p* < 0.05.

## Results

Of 862 patients who underwent SBTKA, eGFR decrease and AKI did not develop in 818 (94.9%). In 44 patients (5.1%), a decrease in eGFR and AKI of different stages were observed in the postoperative period, according to the RIFLE criteria. The mean age and mean operative time in patients without AKI were 71.79 ± 7.65 years, and 83.10 ± 10.38 min, respectively, and they were transfused a mean of 0.97 ± 0.73 units of blood. The mean age and mean operative time in patients with AKI were 74.05 ± 8.41 years, and 110.91 ± 13.26 min, respectively, and they were transfused a mean of 2.14 ± 1.21 units of blood. Among the AKI-negative patients, 8.6% were obese, 17.2% were mildly obese, and 74.1% were normal weight. The same parameters were 22.7%, 31.8%, and 45.4%, respectively, in patients with AKI. In the AKI-negative patients, 12% were ASA I, 62% were ASA II, 31% were ASA III, while there were no ASA IV patients. Among patients with AKI, 13.6% were ASA I, 47.7% were ASA II, 27.2% were ASA III, and 11.3% were ASA IV. 32.7% of the AKI-positive and 36.3% of the AKI-negative patients used ACE inhibitors. In 42 of 44 patients who developed AKI, eGFR returned to normal between the 5th and 14th postoperative days, and there was no permanent damage to kidney functions. However, two patients with renal dysfunction in the preoperative period and in the ASA IV risk group died from multiple organ failure in the 3rd and 6th weeks postoperatively due to increased renal dysfunction. The patients who died in the 3rdand 6th weeks had stage 3 renal failure, and stage 4 loss of kidney function, respectively. We measured the operative time beginning from the incision of the right knee until the closure of layers in the left knee. Except for the death of 2 patients with preoperative renal dysfunction, among 862 patients, the kidney functions of 860 (99.75%) completely returned to normal in the 1st postoperative month. The postoperative urea and creatinine levels of the patients who did not develop AKI increased by approximately 9.2% compared to the preoperative values and eGFR decreased by 8.5%. In the AKI-positive group, urea and creatinine levels increased by approximately 69.4% postoperatively, and eGFR decreased by 41%. The preoperative mean hemoglobin level of all patients was 13.5 mg/dl (12–15.8) while the mean preoperative hematocrit value was 37.5 (34–43). The AKI-positive and negative patients received a mean of 2.14 and 0.97 units of erythrocyte and fresh frozen plasma transfusion (Table 3).

**Table 3:** Predisposing factors in AKI (+) and AKI (−) patients.

		AKI (−) *n*:58		AKI (+) *n*:44		*p*
Age		71.79 ± 7.65		74.05 ± 8.41		0.161^a^
Gender	Male	4	6.90%	5	11.36%	0.431+
	Female	54	93.10%	39	88.64%	
Operative time (min)		83.10 ± 10.38		110.91 ± 13.26		**0**.**0001**^a^
Obesity	Not present	43	74.14%	20	45.45%	**0**.**003+**
	Present	15	25.86%	24	54.55%	
Obesity	Normal	43	74.14%	20	45.46%	–
	MildlyObese	10	17.24%	14	31.82%	**0**.**043+**
	Obese	5	8.62%	10	22.73%	**0**.**028+**
Blood transfusion (Units)		0.97 ± 0.73		2.14 ± 1.21		**0.0001** ^a^
Blood transfusion (Units)	None	15	25.86%	5	11.36%	–
	1–2 Units	42	72.41%	22	50.00%	0.611+
	3–4 Units	1	1.72%	17	38.64%	**0**.**0001+**
Smoking	Smoking (−)	**51**	**87**.**93%**	**33**	**75**.**00%**	**0**.**090+**
	Smoking (+)	**7**	**12**.**07%**	**11**	**25**.**00%**	** **
Preoperative diabetic state	DM (−)	53	91.38%	32	72.73%	**0**.**012+**
	DM (+)	5	8.62%	12	27.27%	
Preoperative hypertension	HT (−)	**36**	**62**.**07%**	**11**	**25**.**00%**	**0**.**0001+**
	HT (+)	**22**	**37**.**93%**	**33**	**75**.**00%**	** **
ACE inhibitor use	ACE inhibitor (−)	39	67.24%	28	63.64%	0.704+
	ACE inhibitor (+)	19	32.76%	16	36.36%	
Anemia	Anemia (−)	**56**	**96**.**55%**	**36**	**81**.**82%**	**0**.**013+**
	Anemia (+)	**2**	**3**.**45%**	**8**	**18**.**18%**	** **
ASA	ASA I + II	40	68.97%	27	61.36%	0.423+
	ASA III + IV	18	31.03%	17	38.64%	

Bold values indicate statistically significant data.

^a^ Independent *t*-test.

^+^ Chi-Square test.

Based on the RIFLE criteria, among patients with AKI, 31 patients (70.4%) had renal risk (R), 9 patients (20.4%) had injury (I), 3 (6.8%) had failure (F), and one (2.2%) had loss of kidney function (L). End-stage renal failure was not observed in any of the patients. Dialysis was not performed on any patient except for one patient with Rifle stage 4(L), who died.

No statistically significant difference was observed between the presence of smoking in the AKI (−) 7 (12.07%) and AKI (+) 11 (25%) groups (*p* = 0.09).

The presence of hypertension in the AKI (+) Group 33 (75%) was found to be statistically significantly higher than in the AKI (−) group 22 (37.93%) (*p* = 0.0001).

The presence of Anemia in the AKI (+) Group 8 (18.18%) was found to be statistically significantly higher than in the AKI (−) group 2 (3.45%) (*p* = 0.013) (Table 4).

**Table 4:** Hemogram and eGFR values of AKI(+) and AKI (−) patients.

		AKI (−) *n*:58	AKI (+) *n*:44	*p* ^a^
BUN	Preoperative	15.83 ± 3.78	16.16 ± 3.01	0.634
	Postoperative	18.00 ± 4.59	23.68 ± 4.56	**0**.**0001**
	*p* ^b^	**0.0001**	**0.0001**	** **
	Pre-and postoperative difference	−2.17 ± 3.87	−7.52 ± 3.66	**0**.**0001**
eGFR	Preoperative	96.96 ± 3.82	94 ± 6.22	**0**.**004**
	Postoperative	88.38 ± 5.06	52.92 ± 16.16	**0**.**0001**
	*p* ^b^	**0.0001**	**0.0001**	** **
	Pre-and postoperative difference	8.57 ± 3.71	41.08 ± 14.13	**0**.**0001**
Hemoglobin	Preoperative	13.47 ± 0.82	13.84 ± 0.86	**0**.**029**
	Postoperative	10.2 ± 0.66	8.85 ± 0.88	**0**.**0001**
	*p* ^b^	**0.0001**	**0.0001**	** **
	Pre-and postoperative difference	3.26 ± 0.69	4.99 ± 0.85	**0**.**0001**

Bold values indicate statistically significant data.

^a^ Independent *t*-test.

^b^ Paired *t*-test.

AKI (−) and AKI (+) groups was observed between the preoperative BUN averages of No statistically significant difference (*p* = 0.634). AKI (+) group were found to be postoperative BUN averages of the statistically significantly higher than those of the AKI (−) group (*p* = 0.0001).

AKI (−) group were found to be Postoperative BUN averages of statistically significantly higher than the preoperative BUN averages (*p* = 0.0001).

AKI (+) group were found to be Postoperative BUN averages of statistically significantly higher than the preoperative BUN averages (*p* = 0.0001).

Multivariate logistic regression analysis was performed with the variables Operative time (min), The presence of obesity, Blood transfusion (Units), Preoperative eGFR, Preoperative Anemia, Preoperative hypertension and Preoperative diabetes which were found to be statistically significant in univariate tests (Table 5).

**Table 5:** Multivariet logistic regression analysis.

	Exp (*B*)	%95 C.I for Exp (*B*)		*p*
		Lower limit	Upper limit	
Operative time (min)	1.15	1.08	2.24	0.0001
The presence of obesity	0.64	0.15	2.71	0.545
Blood transfusion (Units)	3.49	1.69	5.23	0.001
Preoperative eGFR	0.89	0.77	1.05	0.104
Preoperative Anemia	3.13	0.94	6.09	0.081
Preoperative hypertension	0.74	0.18	2.11	0.682
Preoperative diabetes	3.38	0.85	14.51	0.074

While the variables of the presence of obesity (*p* = 0.545), Preoperative eGFR (*p* = 0.104), Preoperative Anemia (*p* = 0.081), Preoperative hypertension (*p* = 0.682) and Preoperative diabetes (*p* = 0.074) were found to be statistically insignificant, the increase in Operative time (min) (*p* = 0.0001) and the increase in Blood transfusion (Units) (*p* = 0.001) were found to be statistically significant. Operative time (min) and Blood transfusion (Units) variables were determined as factors affecting the presence of AKI.

In the differential diagnosis of AKI (+), the area under the ROC curve of operation time was 0.861, and it had a sensitivity of 81.82, a specificity of 78.69, a positive predictive value of 78.45, a negative predictive value of 76.80, an LR (+) value of 3.30 and a cutoff value of >95 min (The risk of developing AKI in a patient with an operation time of >95 min is 3.30 times higher than a patient with an operation time of <95 min) (Table 6).

**Table 6:** ROC analysis.

	Operative time	Blood transfusion (Units)
Area under the ROC curve (AUC)	0.861	0.785
Standard error	0.039	0.047
95% confidence interval	0.778–0.921	0.693–0.860

	Cut off	Sensitivity	Specificity	PPV	NPV	LR (+)
Operation time	>95 (dk)	81.82	78.69	78.45	76.80	3.30
Blood transfusion	>2 (units)	72.73	79.31	72.7	79.3	3.52

**Figure 1:**
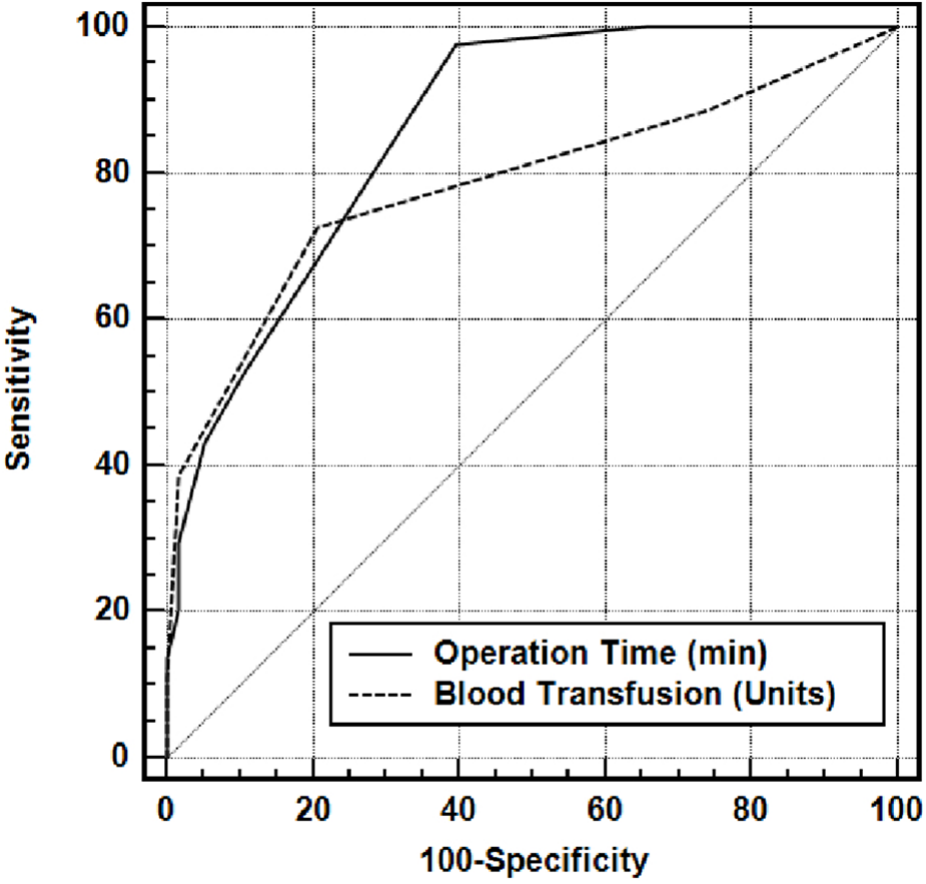
Area under the ROC curve for operative time (min) and blood transfusion (units).

In the differential diagnosis of AKI (+) the area under the ROC curve of **blood transfusion** was 0.785, and it had a sensitivity of 72.73 a specificity of 79.31, a positive predictive value of 72.7 a negative predictive value of 79.3 an LR (+) value of 3.52 and a cutoff value of >2 units (The risk of developing AKI in a patient who received >2 units of blood transfusionis 3.52 times higher than a patient who received ≤2 units of blood transfusion) Figure 1.

## Discussion

AKI is a part of a kidney disease spectrum and characterized by a sudden decrease in kidney functions (20–22). It usually develops after operations with increased bleeding, increases mortality, is reversible, and potentially preventable (23). We found that prolonged operation time in SBTKA, increased amount of blood transfused, preoperative renal disease and high BMI are risk factors for AKI (24, 25).

Extended operation times cause increased bleeding and prolongation of the tourniquet period in elderly patients with comorbidities, thus exposing the surgical site to more hypoxia and deterioration of hemodynamics. Koh et al. stated that long operation, anesthesia and tourniquet times increase the risk of AKI, and a thorough preoperative surgical planning decreases it (26). Jamsa et al. stated that the incidence of AKI almost doubled in operations that lasted 120 min, and more ischemic damage occurred in tissues with long-term tourniquet use (27). In our study, we found that surgical times of longer than 95 min in SBTKA surgery increased the risk of AKI by 3.30 times. The most important reason why the results in our study differ from the data of Jamsa et al.'s study is that Jamsa conducted a Unilateral study and we conducted AKI research on simultaneous bilateral TKA patients (27). The short duration of operation in our simultaneous-bilateral operation significantly reduced the incidence of AKI in our series.

Since the operation is bilateral, both blood loss and postoperative blood transfusion need will be higher. Perioperative blood loss and consequent hypotension are potentially adjustable and preventable factors that can increase renal hypoperfusion and worsen AKI. Koh et al., Abar et al., Rachel et al., and Ekinci et al. reported that a significant decrease in hemoglobin postoperatively due to increased bleeding, and consequently increased blood transfusions increase the risk of AKI (26, 28–30). However, Takeshita et al. stated the opposite (31). In our study, an average of 2.14 units of blood was transfused to AKI-positive patients and 0.97 units to AKI-negative patients, and we found that the risk of AKI increased 3.57 times in patients who received a blood transfusion of more than 2 units. We attribute the most important reason for this increase in risk to the simultaneous bilateral nature of our operation. Therefore, a thorough planning for SBTKA and having high preoperative hemogram values will help reduce the risk of postoperative AKI.

Obese patients are at high risk for hypoperfusion during surgery. The incidence of AKI in obese patients is 65% higher than in non-obese patients. Jafari et al., Abar et al., Jamsa et al., Weingarten et al., Kimmel et al., and Rachel et al. stated that high BMI is a risk factor for postoperative AKI (27, 29, 30, 32–34). In their study of 197 patients, Ali Vial et al. stated that obesity was the most notable risk factor and that comorbidities associated with AKI such as HT and diabetes were common in obese patients (35). On the contrary, Hassan et al. and Takeshita et al. could not detect a relationship between high BMI and AKI (31, 36). In our study, we found that obesity indirectly paved the way for the development of AKI caused by renal hypoperfusion and acute tubular necrosis by prolonging the operation time and increasing the amount of bleeding. If possible, SBTKA should be planned after achieving ideal weight in patients with high BMI, but we believe that it would be appropriate to perform a staged operation if necessary.

In their studies, Abar et al. and Jamsa et al. reported that a high ASA score, Kimmel et al., and Farrow et al. stated that diabetes mellitus, and Ferguson et al., and Kimmel et al. reported that advanced age are risk factors for AKI (27, 30, 34, 37, 38). Takeshita et al. stated that advanced age is not a risk factor for AKI (31). Ali Vial et al. reported that 16.2% of patients developed AKI within the first 72 h post-total joint arthroplasty (TJA), a rate significantly higher than those reported in the literature. The authors identified age, obesity, smoking, and chronic obstructive pulmonary disease as independent risk factors for AKI. They noted that obesity was the most significant risk factor, with a 65% higher incidence of AKI compared to non-obese patients. They also found comorbidities such as hypertension and diabetes to be associated with AKI. Consistent with that study, we also considerobesityto be a risk factor for AKI, whereas factors such as age and smoking are not (35).

Hung et al. examined the incidence of AKI and its risk factors over the past decade in patients undergoing TJA. They found an AKI incidence rate of 0.0051% following TJA. Significant risk factors identified included diabetes, bilateral surgery, high BMI, elevated preoperative blood urea nitrogen, and low preoperative hematocrit levels. They noted that the annual incidence of AKI after TJA remained stable over the past ten years. Our findings are consistent with that study, identifying similar factors as constituting risk for AKI (39).

We do not think that patient age, ACE inhibitor use, comorbidities (excluding diabetic nephropathy), male gender, and ASA score (excluding ASA IV) directly or indirectly affect AKI development. We would like to state that SBTKA surgery should not be preferred in patients with ASA IV risk and diabetic nephropathy. In patients with chronic persistent hypertension, the development of AKI may be triggered by renal vasoconstriction. However, all patients considered for SBTKA should be operated on under normotensive anesthesia during both the preoperative and perioperative periods.

Our study had some limitations, one of the most significant of which was the absence of studies in the literature on AKI, especially in SBTKA. The AKI studies in unilateral TKA were referred to, which had a negative impact on the evaluation of our results. The second limitation is the retrospective nature of our study. It is necessary to consider the fact that other non-surgical factors (NSAID use, smoking, alcohol, IV fluid replacement, etc.) may affect the development of AKI and that prospective studies are needed to include these factors. The strength of our study is that we worked on a large number of patients and reported the experiences of a single surgeon in a single center.

In line with all these details, we hope that our study will shed light on future studies. We believe in the need for more studies revealing the complications of SBTKA compared to unilateral surgery.

## Conclusion

AKI is a multifactorial pathology. We think that proactive management of multiple risk factors can reduce this complication. In our study, long operation time (>95 min), an increased postoperative transfusion need, and preoperative diabetic nephropathy have direct effects, while increased BMI (BMI > 40 kg/m^2^) has indirect effects on AKI development by both prolonging the operation time and requiring extensive dissection. The risk of AKI increases 3.57 times in patients who require 2 or more units of blood transfusion. Considering that the duration of the operation exceeding 95 min for both knees increases the risk of AKI 3.30 times, we recommend that the surgeon perform this operation unilaterally or in stages in operations predicted to last longer. Patient age, ASA scoring (excluding ASA IV), ACE inhibitor use are not risk factors for AKI in SBTKA. The preoperative risk factors of patients to undergo SBTKA should be thoroughly analyzed, and patients with high AKI risk should have their preventable risk factors optimized. We find it more appropriate for patients with unavoidable risks to undergo staged BTKA.

## Data Availability

The original contributions presented in the study are included in the article/Supplementary Material, further inquiries can be directed to the corresponding author.
